# Antiinflammatory Effect of Phytosterols in Experimental Murine Colitis Model: Prevention, Induction, Remission Study

**DOI:** 10.1371/journal.pone.0108112

**Published:** 2014-09-30

**Authors:** Rita Aldini, Matteo Micucci, Monica Cevenini, Romana Fato, Christian Bergamini, Cristina Nanni, Massimiliano Cont, Cecilia Camborata, Silvia Spinozzi, Marco Montagnani, Giulia Roda, Antonia D'Errico-Grigioni, Francesca Rosini, Aldo Roda, Giuseppe Mazzella, Alberto Chiarini, Roberta Budriesi

**Affiliations:** 1 Department of Pharmacy and Biotecnology, University of Bologna, Bologna, Italy; 2 Department of Medicine and Surgery, University of Bologna, Policlinico S Orsola, Bologna, Italy; 3 Department of Nuclear Medicine, Azienda Ospedaliero-Universitaria di Bologna Policlinico S.Orsola-Malpighi, Bologna, Italy; 4 Department of Chemistry “Giacomo Ciamician”, University of Bologna, Bologna, Italy; 5 DIMES Department, University of Bologna, Policlinico S Orsola, Bologna, Italy; University of South Carolina School of Medicine, United States of America

## Abstract

Phytosterols, besides hypocholesterolemic effect, present anti-inflammatory properties. Little information is available about their efficacy in Inflammatory Bowel Disease (IBD). Therefore, we have evaluated the effect of a mixture of phytosterols on prevention/induction/remission in a murine experimental model of colitis. Phytosterols were administered *x os* before, during and after colitis induction with Dextran Sodium Sulfate (DSS) in mice. Disease Activity Index (DAI), colon length, histopathology score, ^18^F-FDG microPET, oxidative stress in the intestinal tissue (ileum and colon) and gallbladder ileum and colon spontaneous and carbachol (CCh) induced motility, plasma lipids and plasma, liver and biliary bile acids (BA) were evaluated. A similar longitudinal study was performed in a DSS colitis control group. Mice treated with DSS developed severe colitis as shown by DAI, colon length, histopathology score, ^18^F-FDG microPET, oxidative stress. Both spontaneous and induced ileal and colonic motility were severely disturbed. The same was observed with gallbladder. DSS colitis resulted in an increase in plasma cholesterol, and a modification of the BA pattern. Phytosterols feeding did not prevent colitis onset but significantly reduced the severity of the disease and improved clinical and histological remission. It had strong antioxidant effects, almost restored colon, ileal and gallbladder motility. Plasmatic levels of cholesterol were also reduced. DSS induced a modification in the BA pattern consistent with an increase in the intestinal BA deconjugating bacteria, prevented by phytosterols. Phytosterols seem a potential nutraceutical tool for gastrointestinal inflammatory diseases, combining metabolic systematic and local anti-inflammatory effects.

## Introduction

Recently, there has been a raising an interest towards essential oils and lipid-soluble bioactive compounds from various natural sources among scientists.

Phytosterols and phytostanols are plant derived sterols, structurally related to cholesterol. Phytosterols differ from cholesterol only for the side chain, which contains a methyl (ampesterol) or ethyl (β-sitosterol and stigmasterol) group [1], while phytostanols lack the Δ^5^ double bond in the B ring. Saturated forms of plant sterols (phytostanols) are less represented in nature than the unsaturated forms (terols) [2]. Woods and vegetables are the main sources for pharmaceutical phytosterols [3]. Phytosterols are natural components of human diets, and they are found, mostly, in vegetable oils, cereals, fruits and vegetables, while common dietary sources of phytostanols are corn, wheat, rye, and rice [4]. β-sitosterol, the main sterol of several plant species, has been shown to exert anti-inflammatory, anticancer, antispasmodic, antioxidant, and antidiabetic activities [5] [6] [7] [8]. Phytosterols have been shown to determine hypocholesterolemic effect, in normal subjects and hypercholesterolaemic subjects, without affecting high-density lipoprotein cholesterol and triglycerides levels [9]. There is good experimental support for the traditional idea that phytosterols act within the intestinal lumen where they displace cholesterol and balance whole body cholesterol absorption and excretion [10]. However, the exact molecular mechanisms responsible for cholesterol-lowering activity of plant sterols/stanols is likely a complex interplay of multiple processes, among which the so called transintestinal cholesterol excretion [11].

In addition to their cholesterol lowering efficacy, experimental [12] [13] [14] [15] and clinical [16] [17] studies have suggested that plant sterols have anti-inflammatory properties. The anti-inflammatory effect of plant sterols has been addressed mainly to atherosclerosis, where inflammation plays a major role in the process of atherogenesis [18]. However, the anti-inflammatory effect [6] [19] [20] of phytosterols is independent of the hypocholesterolaemic effect [13] [21] [22].

The inhibition of inflammatory processes is essential to control or prevent various diseases, inflammatory bowel disease (IBD), This disorder, which mainly refers to Crohn's disease and Ulcerative Colitis, is a disordered interplay of genetic, microbial and environmental factors leading to the activation of the mucosal immune and non-immune response, resulting in active inflammation and tissue destruction. This disorder, which refers mainly to Crohn's disease and Ulcerative Colitis, has increased, in last years, in several countries. Few studies are available about the effects of phytosterols in experimental model of colitis such as trinitrobenzene sulfonic acid (TNBS) or dextran sodium sulfate (DSS) that has usually been used to generate an animal model which closely mimics Crohn's disease or Ulcerative Colitis, respectively[23] [24]. The DSS mouse model is a colitis model resembling the colitis occurring in patients with inflammatory bowel diseases such as Crohn's disease and ulcerative colitis. These two clinical entities manifest with a similar pattern of symptoms when the disease is expressed in the colon. However, the pathophysiology of both disorders is poorly understood and seems to be different. Many studies have shown the anti-inflammatory effects of phytosterols[14] of β-sitosterol [25] sterol guggulsterone [26] [27] and phytosteryl ferulates [28] in experimental IBD.

The available literature shows a relatively high degree of variability, making it very difficult to reach a solid conclusion about phytosterols anti-inflammatory effect in IBD [21] [29] [30]. This variability may be due to the different mode of administration and the presence of other phytochemicals. So far substantiated clinical outcomes are still lacking [21]. Generally, IBD treatment is based on anti-inflammatory drugs or immunosuppressive drugs such as 5-aminosalicylic acid (5-ASA) and 6-mercaptopurine respectively and anti-TNFα drugs [31]. Most of these drugs show severe side effects that limit their use [32] [33].

Various natural products have been shown to safely suppress pro-inflammatory pathways and control IBD in *in vivo* and/or *in vitro* studies [34]. However the health claims of plants, vegetal extracts, natural substances, food supplements and functional foods have to be supported by scientific data and approved by the Food Safely Authorities such as EFSA (European Food Safety Authority) and U.S. FDA (Food and Drug Administration). The investigations so far reported on the anti-inflammatory effect of phytosterols in experimental IBD are either acute observations, spanning an overall 72 hours period from induction of colitis and sacrifice of the animals after 24 hours treatment [14] [or preventive and therapeutic short time studies spanning over a few days [26] [27] or longer experiments, but not extending beyond the colitis induction period [28]. To our knowledge, a longitudinal preventive and therapeutical study about the role of phytosterols in the prevention/induction and remission of inflammation in IBD and has not been reported, neither a study about the possible role ofphytosterols on the IBD related motility disorders.

Aim of the present investigation was to evaluate the effect of a β-sitosterol, campesterol, stigmasterol and brassicasterol combination on prevention, and remission of clinical symptoms and mucosal healing in a mouse model of DSS induced colitis. Furthermore, we evaluated the effect of the phytosterols combination towards colon, ileum and possibly gallbladder functional motility disorders induced by DSS [35].

In addition, in order to longitudinally monitor inflammation in the same animals and to evaluated the response to Phytosterols treatment we used [2-deoxy-2-[^18^F]Fluoro-D-glucose(FDG)] micro-Positron Emission Tomography (^18^FDG-microPET) which has been successfully applied in murine models of chemically induced colitis [36] [37].

## Materials and Methods

### Animals

Sixty male Balb/c mice (8 weeks old, 22–25 g b.w.) (Charles Rivers Laboratories, Calco, LC, Italy) were enrolled. A higher number of animals than required were recruited in order to compensate for the drops out in the course of the experiments, due to the severity of colitis compelling to stop the experiments, according to the Recommendations of the Ethical Committee of the University of Bologna for Animal Experiments.

### Ethical Statements

The work has been conducted according to the guidelines set forth by EEC Directive 86/609 on the care and use of experimental animals. The protocol for the induction of colitis was approved by the Institutional Ethics Committee of the University of Bologna (Protocol 22/03/10). All studies involving animals are reported in accordance with the ARRIVE guidelines [38] [39]. Animals were housed in a controlled environment (22–24°C), maintained on a standard 12-h light/dark cycle (lights on at 07.00 h) and had free access to food and water throughout the study. The total number of animals used in our study was 60 (20 control mice and 40 mice with colitis). Three mice died during the colitis induction; thus, the number of animals used in the experiments was 57. The authors confirm that the protocol was specifically approved by the “Comitato Etico Scientifico per la Sperimentazione Animale” of the University of Bologna and transmitted to the Ministry of Health(artt. 7-8-9 D.Lgs.116/92).

### Experimental protocol

**Figure 1:**
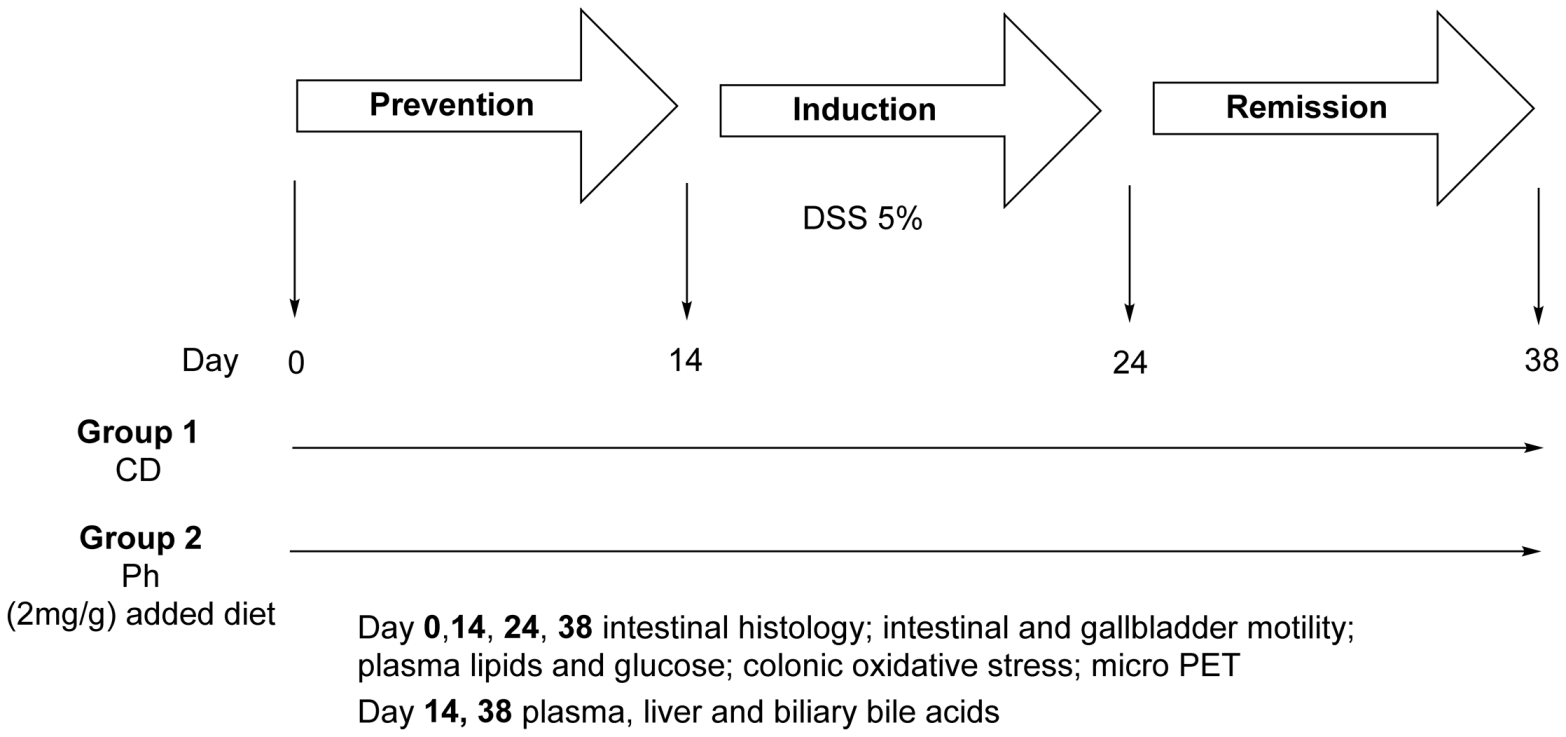
Overview of experimental design. Since at time points indicated by arrows 3–5 animals of each group were sacrificed, a total of 10 mice in the Ph group and 6 mice in the CD group (4 died) for each group completed the study. Mice receiving Ph for 14 days (day 0–14) were used to examine the Prevention of DSS induced colitis by Ph and compared with mice consuming CD. Mice receiving DSS from day 14 to day 24 and Ph were used to examine the response to Ph during the acute phase. Mice receiving Ph over 10 days after DSS was stopped and substituted for with water were used to evaluate the remission induced by Ph.

A prevention/induction/remission study has been conducted as follows: animals were fed either usual commercial control diet CD (CD group) or the same diet enriched with a phytosterols (Ph) preparation (Solgar, Via Prima Strada, 23 int. 3 - 35129 Padova – Italy). For details about composition of the nutraceutical see File S1: About administered Phytosterols (Figure S1 and Table S1 in File S1) (Ph group) throughout the study. Experimental colitis was induced by giving mice drinking water ad libitum containing DSS (MP Biomedicals, Solon; OH, USA; m.w. 36.000–50.000) (5g%, v/w), w/v) from day 14 to day 24 in both groups Colitis was induced starting at day 14until day 24 by oral administration of DSS (5g%, v/w), w/v) in drinking water in both groups. Thereafter, mice either continued the usual diet or the Phytosterols enriched diet over 14 days. Throughout the experiment, the Disease Activity Index (DAI) was evaluated every other day. Before, during and after colitis induction (5 and 10 day) and after days 14 mice were fed with either phytosterols or control diet, 3–5 mice of each group were sacrificed and the following parameters were evaluated: colon length, intestinal histology, ileal, colonic and gallbladder motility, ileal and colonic oxydative stress. At the beginning of the study, after 14 days either CD or Ph added diet, after 10 days DSS administration and 14 days after stopping DSS (each group continuing its own diet) ^18^F-fluorodeoxyglucose (^18^F-FDG) micro Positron Emission Tomography (microPET) was performed in at least 6 animals from each group. At the same time intervals, 3–5 mice of each group were sacrificed and the intestinal histology, ileal, colonic and gallbladder motility, ileal and colonic oxidative stress, plasma, lipids and glucose were evaluated. Plasma, liver and biliary bile acids (BA) were studied immediately after mice were fed with CD and Ph and at the end of the study (Figure 1).

### Phytosterols Administration

Mice were on a 4RF21diet (Mucedola S.r.l., Milan, Italy). The 4RF21 complete food was added with Phytosterols at a final concentration of 2 g/kg. This provides an average dose of 400 mg/kg/day for mouse [40]. For Details, see File S2: Details for Phytosterols Administration]

### Induction of DSS Colitis

Acute colitis was induced by DSS administration according to Waldner [41] and Wirtz [42]. For Details see File S3: Details for DSS Induction of Colitis]. Stools were evaluated for consistency and presence of blood.

### Experimental Procedure

Twenty four hours before the experiments, food supply was withdrawn, while water was maintained *ad libitum*. The animals were sacrificed by cervical dislocation. Immediately before the sacrifice, blood was withdrawn by cardiac puncture and collected in heparinized tubes and plasma was frozen at −80°C for BA analysis. The abdomen was opened by median laparotomy and exposed. The gallbladder was visualized, the cystic duct tied and dissected from the common bile duct. Bile was removed with an insulin syringe (29G) and stored at −80°C until BA analysis. The removed gallbladder was immediately used for motility studies.

The portion of jejunum, immediately after the ligament of Treitz (ligamentum suspensoriun duodeni) was removed (1.5 cm) and retained for histological examination. A 1.5 cm length segment of the terminal ileum, immediately proximal to the ileo-cecal valve and a 2.5 cm region of the distal colon were identified, gently flushed with Krebs solution to remove fecal residues and dissected into two parts: one part was placed in 10% formalin for subsequent histological analysis, a second was retained for immediate in vitro motility studies (see below) or frozen at −80°C for oxydative stress analysis. A liver sample (2g) was collected and frozen at −80°C for BA analysis.

Before and after the study was completed, total cholesterol, HDL cholesterol, triglycerides and glucose plasma levels were measured in samples taken by cardiac puncture respectively from 6 control animals, 6 animals receiving phytosterols and 6 animals receiving control diet, 14 days after DSS was stopped and each group had continued its own diet. triglyceride, HDL-cholesterol, total cholesterol and glucose concentrations were measured using Dimension RxL Max system (Siemens Healthcare Diagnostics, Newark, DE, USA), following manufacturer's instructions. For each mouse a plasma specimen volume of 100 µl was tested.

### Assessment of inflammation

Symptoms and colon length were evaluated as previously described [36]. For details, see File S4: Details for Assessment of Inflammation and Table S4 in File S4).

#### Evaluation of disease activity index (DAI)

DAI was calculated for each animal as described by Fitzpatrick et al.[43] Briefly, body weight, stool consistency and stool blood were recorded every three days. DAI was determined by combining scores of body weight loss, stool consistency and stool blood. The average of the three values constituted the DAI. Body weight loss was calculated as the percent difference between the initial weight (day 0) and the body weight at each time point. Stools blood was determined using the occult blood test kit (Shionogi & Co. Ltd, Osaka, Japan). Parameters investigated for the evaluation of the Disease Activity Index are presented in Table 1.

**Table 1:** Parameters investigated for the evaluation of the Disease Activity Index.

Score	Weight Loss	Stool Consistency	Blood in Stool
**0**	No	Normal	No
**1**	1–5%	Normal	+
**2**	6–10%	Very soft but formed	++
**3**	11–15%	Liquid	+++
**4**	>15%	Liquid	Gross rectal bleeding

#### ^18^F-FDG micro PET

Mice were housed under conditions of controlled temperature (24°C–26°C). Imaging studies were performed using a small animal PET tomograph (GE, eXplore Vista DR) using ^18^fluoro-deoxyglucose (FDG) for glucose metabolism and a CT system (microCT eXplore Locus, GE). ^18^F-FDG was synthesized in an automatized module (FASTlab, GE Healthcare). Both PET and the CT system were designed for small animals. Synthesis and quality control of ^18^F-FDG were routinely performed in the Technological Unit of the S. Orsola Hospital in Bologna. Animals were fasted 16 hours and water was allowed *ad libitum* before the scanning procedure, and colon was washed with phosphate buffered saline (PBS) solution, as for colonography [44]. ^18^F-FDG-PET was carried out as follows: mice were anaesthetized (Sevofluorane 3–5% and oxygen 1 l/min) and injected with 20 MBq of ^18^F-FDG, in a volume of 0.1 ml, via the tail vein with an insulin syringe (24 gauge). Mice were subsequently allowed to wake up for the uptake period (60 min), during which they were allowed to move freely. The residual dose was measured to verify the effective dose injected. Finally, anesthesia was induced a second time in the same way for performance of the scan. Each anesthetized animal was placed on the scanner bed in the supine position. Images were acquired for a total acquisition time of 15 min. As the axial field of view was 4 cm, one bed position was sufficient to cover the parts of interest. Once the scan had been completed, the gas anesthesia was stopped and the animal was kept in controlled temperature conditions until recovery. ^18^F-FDG-PET images were reconstructed iteratively (3D FORE 2D OSEM function) with the software of the PET tomograph (GE, eXplore Vista DR), than read in three planes (axial, sagittal and coronal). Scan was considered positive when regions of increased (ROIs) of ^18^F-FDG uptake were present in sites consistent with the site of inflammation. Semi-quantitative analysis (maximum standardized uptake time, SUV max) of tracer uptake was calculated in the hottest area within the colon as follows:
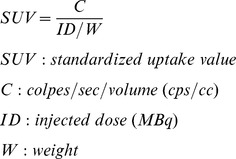
Data are presented as means ± SE of n experiments. Figures were created using GraphPad Prism version 5.0 software [45]

### Histology

*Histology and histological score* For details, see File S4 and Table S4 in File S4.

### Determination of Plasma lipids and glucose

Triglyceride, HDL-cholesterol, total cholesterol and glucose concentrations were measured using Dimension RxL Max system (Siemens Healthcare Diagnostics, Newark, DE, USA), following manufacturer's instructions. For each mouse a plasma specimen volume of 100 µl was tested.

### Determination of Plasma, Liver and Biliary Bile Acids

Total BA in plasma, liver and bile were determined by HPLC-ES-MS/MS [46]. For details, see File S5: Details for Determination of Plasma, Liver and Biliary Bile Acids).

### Oxydative stress evaluation in the ileal and colonic tissues

Thiobarbituric acid (TBA) assay of malondialdehyde (MDA): colon and ileum were homogenized in distilled water by means of an ultra-turrax homogenizer. Quantification of TBA reactive substances (TBARS) was carried out as described by Buege and Aust [47]. Extraction and quantification of Coenzyme Q: colon and ileum from mice were homogenized in distilled water by means of an ultraturrax homogenizer. Extraction of coenzyme Q from homogenized colon and ileum tissues was performed as described by Takada et al. [48]. Quantification of CoQ9 was performed by HPLC analysis. 20 µL of ethanolic extract was chromatographed on a C18 column (Kinetex, Phenomenex, 2.6 µm, 100×4.6 mm), using a mobile phase consisting of ethanol: water (97∶3, v/v) at a flow rate of 0.4 ml/min. The concentrations of CoQ9 were obtained by comparison of the peak areas with those of standard solutions.

### Functional *in vitro* studies

For all assays male Balb/c mice (8 weeks old, 25–30 g b.w.) were used. The gallbladder was opened and washed in Krebs solution. The terminal portion of ileum (immediately proximal to the ileo-caecal junction) was cleaned, and 1 cm segments were used. One cm segmentof the distal colon was transected and the mesenteric tissue was removed. The tissues were rinsed and mounted in 15-ml organ bath containing appropriate solution (see below) Each segment was mounted longitudinally under a resting tension of 0.5 g (gallbladder) or 1 g (others). Tissues were allowed to equilibrate for 60 min during which time the bathing solution was changed every 10 min.

Ileum: Tyrode solution of the following composition (mM): NaCl, 145; KCl, 2.6; CaCl_2_•2H_2_O, 1.5; MgCl_2_•6H_2_O, 0.73; NaH_2_PO_4_•2H_2_O, 0.33; NaHCO_3_ 4.8; glucose 11.1; distal colon: Krebs solution of the following composition (mM): NaCl, 119; KCl, 4.5; CaCl_2_•2H_2_O, 2.5; MgSO_4_•7H_2_O, 2.5; KH_2_PO_4_•2H_2_O, 1.2; NaHCO_3_ 25; glucose 11.1; gallbladder: Krebs-Henseleit solution of the following composition (mM): NaCl, 118; KCl, 5.9; CaCl_2_•2H_2_O; MgSO_4_•7H_2_O, 1.2; Na_2_HPO_4_•2H_2_O, 1.0; NaHCO_3_ 25; glucose 10. The physiological salt solution (PSS) was buffered at pH 7.4 by saturation with 95% O_2_ – 5% CO_2_ gas, and the temperature was maintained at 35°C. After an equilibration period (60–90 min), the tissues were used to test spontaneously and carbachol induced contraction.

#### Basal spontaneous motor activity

Under appropriate tension each muscle strip was equilibrated for 1 h and the basal spontaneous motor activity was recorded.

#### Cholinergic activity

Appropriate tension was applied to each muscle strip, then equilibrated for 1 h. Concentration-response curves were constructed by cumulative addition of the agonist (carbachol). Cumulative concentration-response curves to Carbachol (CCh) were constructed by cumulative addition of the agonist. The concentration of agonist in the organ bath was added only after the response to the previous addition had attained a maximal level and remained steady. Contractions were recorded by means of displacement transducer (FT. 03, Grass Instruments, Quincy, MA) using Power Lab software (ADInstruments Pty Ltd, Castle Hill, Australia). Concentration–response curves to agonist were obtained at 30 min intervals, the first one being discarded (ileum and distal colon) and the second one used as control. A new concentration-response curve to agonist was obtained after incubation with the antagonist (Atropine). Tension changes were recorded isotonically for ileum and distal colon. In all cases, parallel experiments in which tissues did not receive any antagonist were run in order to check any variation in sensitivity. It was always verified that the EC_50_ values for the agonist in tissues receiving only the solvent were not significantly different (P>.05) from the control values. In all other cases experiments were discarded.

For basal spontaneous motor activity the tracing graphs of spontaneous phasic contractions were continuously recorded. At the end of experiments, the amplitude of spontaneous phasic contraction (the difference between the basal level and the peak value was measured). The amplitude of the peach (milligrams) and the frequency (cycle per minute) as measured. Functional activity of CCh and antagonism of atropine *vs* CCh induced contraction, was determined in gut segments taken from different groups of mice. The biological results of agonist CCh was expressed as pEC_50_ values. The antagonism activity of atropine against CCh was expressed as p*A*_2_ values determined from Schild plots [50] constrained to slope –1.0 [51], as required by theory.

### Statistical Analysis

#### Evaluation of disease activity index (DAI); Determination of Plasma lipids and glucose; Determination of Plasma, Liver and Biliary Bile Acids

Data are presented as means ± SD of n experiments Differences between mean values were performed by using a two-tailed unpaired Student's *t* test for continuous data. P value less than.05 was considered significant.

*^18^F-FDG micro PET*; *Functional in vitro studies*: Data are presented as means ± SE of n experiments. Differences between mean values were performed by using a two-tailed unpaired Student's *t* test for continuous data. P value less than.05 was considered significant.

#### Oxydative stress evaluation in the ileal and colonic tissues

Statistical analysis was performed by one way ANOVA test.

### Chemicals

See File S6: Details for Chemicals.

## Results

### Assessment of acute colitis and evaluation of the response to Phytosterols

#### Symptoms and colon length

**Figure 2:**
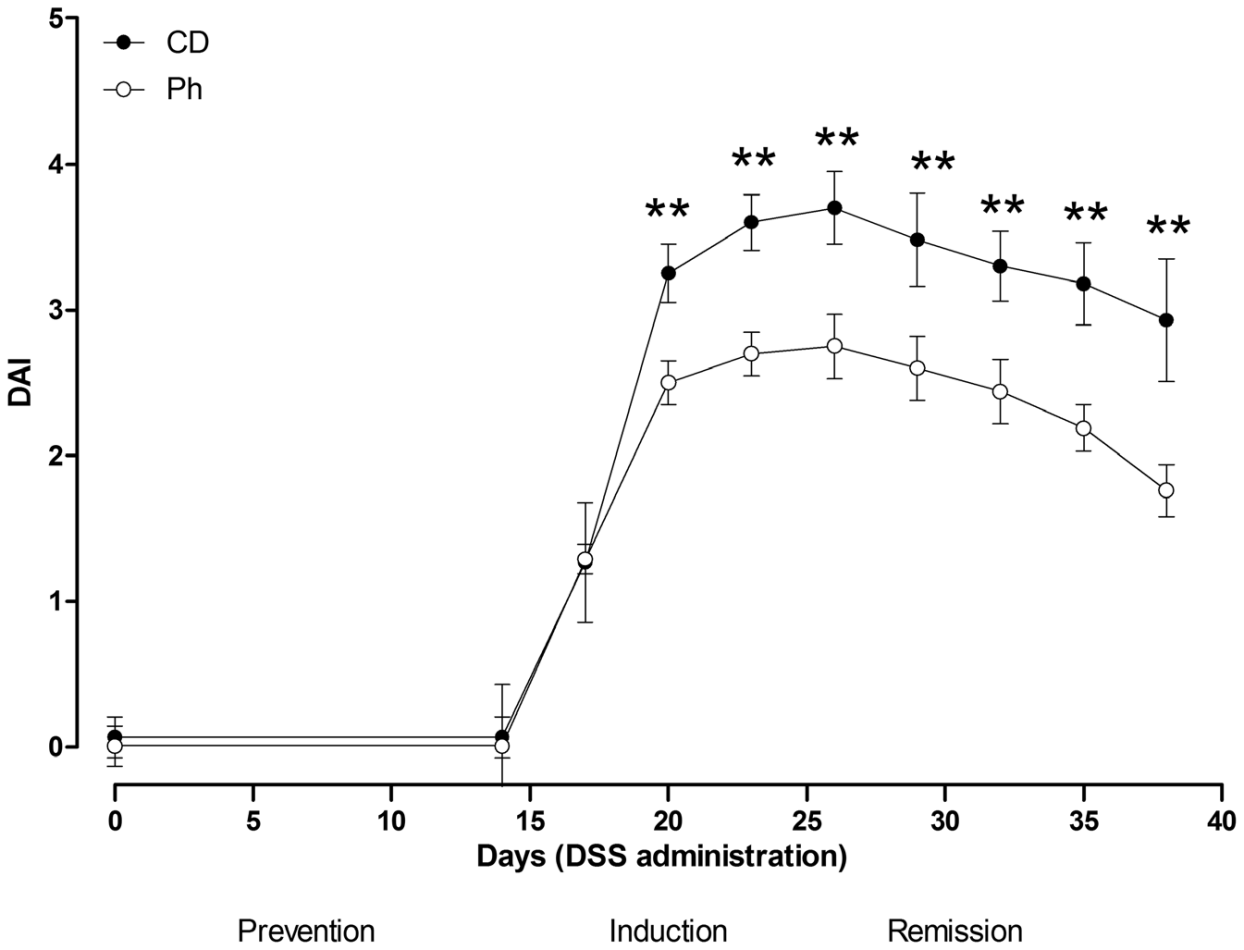
Assessment of acute colitis and evaluation of the response to Ph. Disease Activity Index. DAI evaluation throughout the study in prevention (days 0–14), induction (days 14–24) and remission (days 24–38) in CD and Ph mice respectively (*P<.05. **P<.01). DSS (5% v/w) was added to tap water. DAI was evaluated: from day 1 to day 14 on 27 CD and 27 Ph mice respectively; since at day 14, 9 mice of the CD group and 9 mice of the Ph group were sacrificed, from day 14 to day 24, DAI was evaluated on 18 mice of each group; since at day 24, 9 mice of the CD group and 9 mice of the Ph group were sacrificed, from day 24 to day 38, it was evaluated on 9 mice of the CD group and 9 mice of the Ph group.

The clinical symptoms and histopathological changes found were consistent with acute colitis. In contrast to C57BL/6 mice where a short 5-day exposure of DSS is sufficient to induce progressive chronic colitis, at the same schedule, BALB/c mice develop acute colitis, which resolves after DSS removal [24]. In the present investigation we have used dose of DSS higher than the usual dose [24], to induce more severe acute histological damage and a longer reparation time, in order to closely evaluate over time the reparation or healing process. The food daily intake was similar in the usual CD and in the Ph added diet animals. A combinatorial DAI, considering body weight loss, stool consistency and stool blood, was used to evaluate the activity of the disease and the response to treatment. DAI during acute colitis induction and remission is reported in Figure 2.

Ph administration did not prevent the onset of colitis, but were effective in significantly decreasing severity of colitis and improvingremission. Appearance of fresh emitted stools varied from frank bloody diarrhea to amorphous moist pellets with blood. When a mouse didn't show abnormal feces for at least 4 days before the sacrifice it was discharged. At the end of the induction period, the animals that did not develop loss of at least 10% of the initial weight were discharged. A mortality rate of 4% and 0% was observed respectively in the control diet animals and in the animals assuming Ph. At the autopsy, all the deceased animals showed marked distention of the abdomen and colon with massive endoluminal bleeding and in one case perforation of the colon.

Length of the colon: in CD mice, the colon length was 7.6±0.2 cm; in Ph group, the colon length was not different (7.6±0.3 cm); after acute colitis induction, the colon length was reduced to 6.1±0.2 cm (*P*<.01) and to 6.4±0.2 (*P*<.01) respectively in CD and Ph group (*P*<.05). When mice were fed phytosterols over 14 days after stopping DSS, an increase in colon length (7.4±0.2 cm) (*P*<.01) was observed. In CD mice, 14 days after DSS, the colon length was also increased (6.9±0.1 cm) (*P*<.01). By the end of the study, the colon of Ph fed mice was significantly longer than the colon of CD mice (*P*<.01). Of note, shortening of the colon was completely reversed by Ph.

#### ^18^F-FDG small animal PET

**Figure 3:**
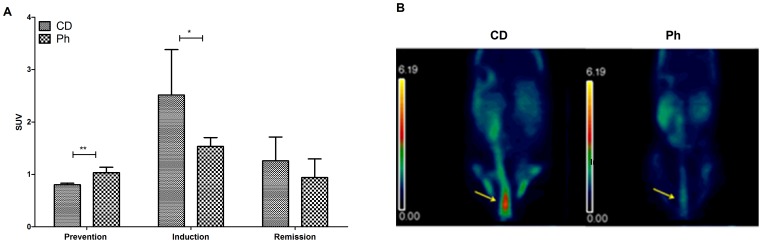
Assessment of acute colitis and evaluation of the response to Ph. ^18^F-FDG micro PET. A. Quantification of ^18^F-FDG uptake (SUV) in the distal colon of Balb/c mice fed with CD and Ph. Analysis were repeated on the same mice at day 14^th^, after CD and Ph (respectively n = 5; n = 5, **P = .0014), at day 24^th^, the last of the ten days of DSS colitis induction (CD n = 5; Ph n = 5, ns), at day 38^th^, fourteen days after the end of the DSS administration (CD n = 4; Ph n = 5, * *P*<.05). B. Representative PET image showing different ^18^F-FDG accumulation in the distal colon (yellow arrows) of Balb/c mice fed with CD (left image) and Ph (right image) at the end of induction. On the left of each images the SUV chromatic scale, from 0.00 to 6.19, is reported.

Regions of interest (ROIs) were selected in the distal colon of coronal sections of the 2D reconstructed images (Figure 3B yellow arrows), where the activity was found higher than in other segments of colon. Activity, measured in cps/cc (events/seconds/volume) necessary to calculate SUV (see Materials and Methods section), was measured for each group of mice, at the end of the prevention, induction and remission period (Figure 1). Surprisingly, at the end of the prevention period the activity was significantly higher in the Ph group (n = 5) than in the CD group (n = 5) (Figure 3A), showing higher activation of the organ in the Ph fed mice. This latter finding was not considered pathological, since there is often low-grade activity in the small colon, due to a combination of physiological factors, including normal intraluminal activity, lymphoid tissue uptake, and smooth muscle uptake [52]. Throughout the study the distal colonic PET signal was consistent with the histological findings.

In particular, during colitis induction, FDG-microPET quantification mimicked the time course of DAI, MDA and histological inflammation. At the end of the induction period, ^18^F-FDG uptake increased in the two groups compared with the pretreatment period, and was significantly higher in the CD group than in Ph group(*P*<.05). Finally, 14 days after DSS suspension, ^18^F-FDG uptake decreased and it was lower in the distal colon of Ph fed mice, compared to CD fed mice. In all the distal\ colonic PET signal with the expected histological inflammation score at these time points. No ^18^F-FDG uptake was observed in the small intestine, differently from Brewer [36], which is consistent with the DSS model of colitis that does not show clinical or histological signs of involvement of the small intestine. In Figure 3B a representative PET image showing the different ^18^F-FDG accumulation in the distal colon of a mouse fed CD (left image) and Ph (right image) at the end of induction is reported. The representation of the semi-quantitative PET analysis (Figure 3A) shows, in the induction, a difference of about 60% of the maximum SUV parameter measured in the two groups (CD; Ph) when the image (Figure 3B) was taken (SUV: CD *vs* Ph = 2.5 *vs* 1.5 = 1.6).

### Evaluation of oxidative stress level in intestinal tissues

**Figure 4:**
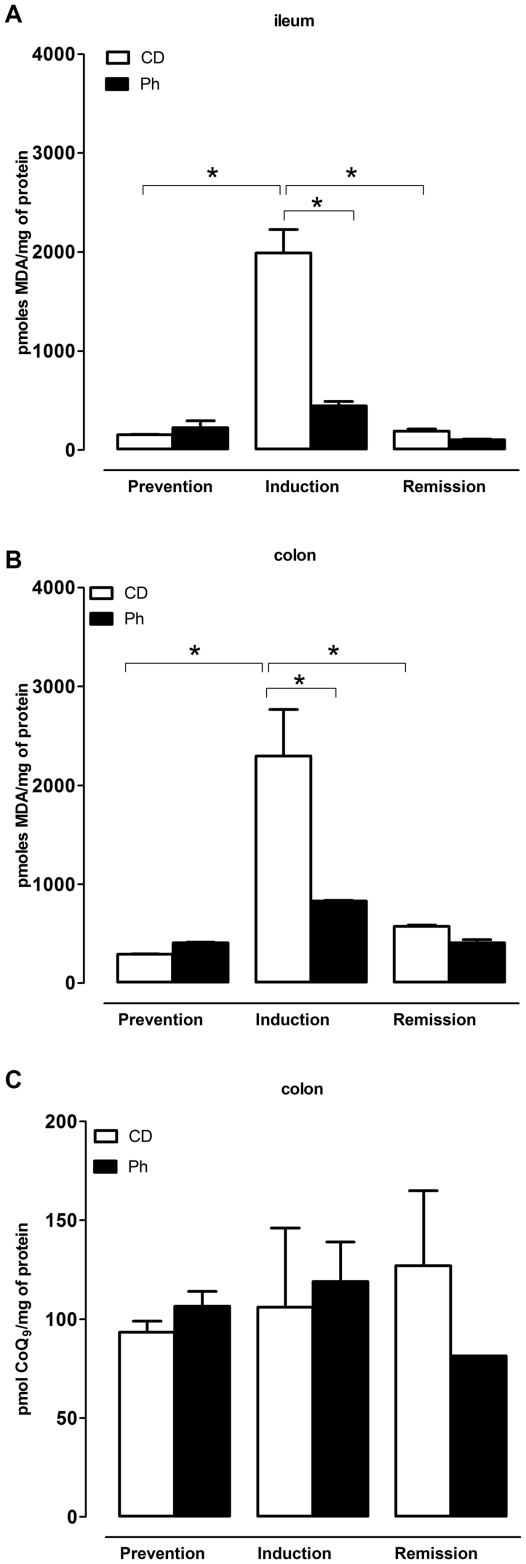
Assessment of acute colitis and evaluation of the response to Ph. Oxydative stress. MDA equivalents in ileum (A) and colon homogenates (B) White bars refer to CD and black bars refer to Ph treatment in the prevention, induction and remission period. In both ileum and colon, a strong oxidative stress is observed in the CD group in the induction phase, shown by a significant increase in MDA equivalents, prevented by Ph treatment (n = 4, *P<.001). The increase is significantly higher than in the prevention and remission phases (n = 4, *P<.001). C. Coenzyme Q_9_ levels in colon homogenate measured by HPLC. The ubiquinone content is not significantly different in all experimental conditions. Each tissue was analyzed in triplicate. Statistical analysis was performed by one way ANOVA test.

MDA levels in colon and ileum specimens are reported in Figure 4. During prevention, MDA levels were similar both in the ileum and in the colon in CD and Ph mice (Figure 4A and B). After DSS administration (induction), MDA ileal and colonic levels increased in both groups, but they were significantly higher in the CD group (Figure 4A and B). Duringremission, MDA levels decreased, but remained significantly higher than before DSS administration in the colon of CD mice (Figure 4A and B). In Ph mice, physiological MDA levels were restored. CoQ9 content in intestinal tissues was not modified by Ph pre-treatment throughout all the study both in colon (Figure 4C) and ileum (data not shown).

### Histology

**Figure 5:**
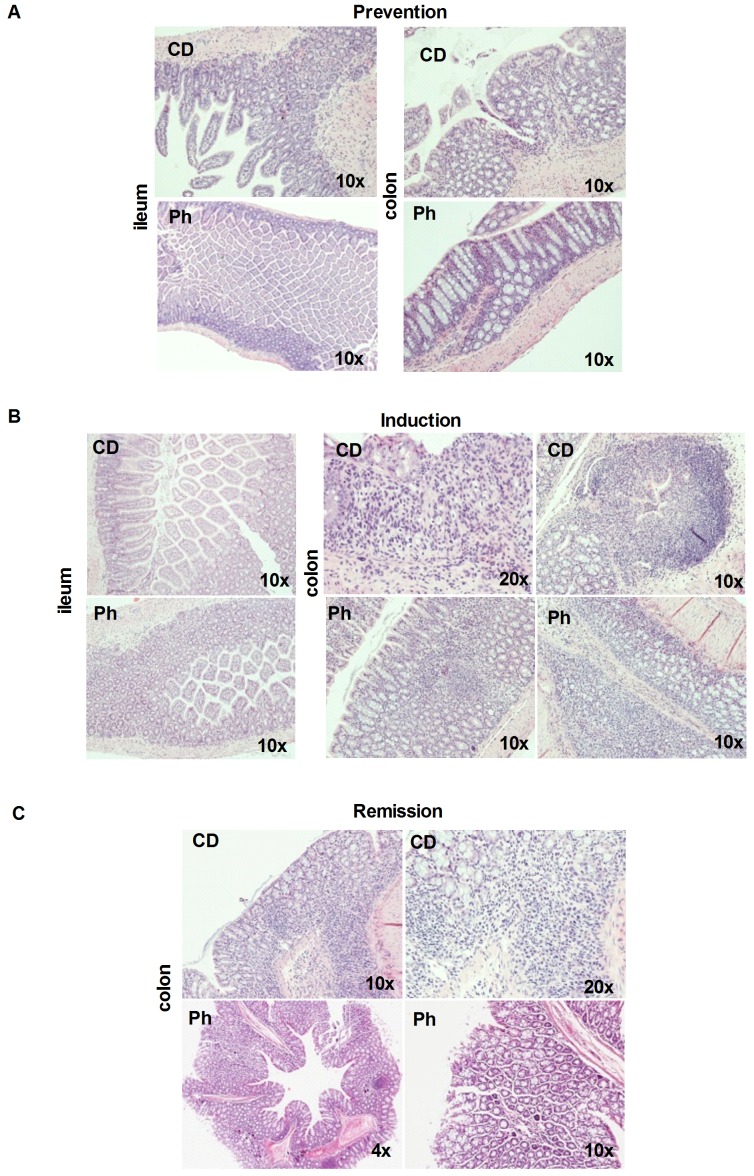
Assessment of acute colitis and evaluation of the response to Ph. Histology. Prevention: normal ileum (Left) and colon (Right) in CD and Ph mice. (H&E,4x). Induction: colonic sections from ileum and colon from CD and Ph. The ileum is normal in both groups. Colon. Upper panel. Left: mucosal ulceration with linfoplasmacytic and neutrophils infiltrate extending to the entire mucosa (H&E, 20 x). Right: cryptic abscess (H&E, 10 x). Lower panel: colonic sections from Ph mice at the end of the induction period. Left: The mucosa and sub-mucosa show a moderate inflammatory infiltrate with lymphocytes and neutrophils (H&E, 10 x). Right: follicular inflammation. (H&E, 10 x). Remission. Upper panel: colonic mucosa specimens. Left: inflammatory infiltrate (H&E, 4x). Right: inflammatory infiltrate with lymphocytes and neutrophils (H&E, 20 x). Lower panel: colonic mucosa specimens. Left: minimal focal lymphocytes infiltration (H&E, 4x). The same at higher magnification (H&E, 10x).

In CD fed mice, DSS induced a severe acute colitis. DSS produced higher scores (Table of Score in (Table S4 in File S4)] of microscopic damage in the CD group (3–4) than in the in Ph group (2–3) when assessed at the end of colitis induction. In the prevention, the histological analysis of control mice and Ph pretreated mice revealed no sign, or only a very low level, of leucocyte infiltration into the colon, with preserved epithelial integrity (Figure 5A) In contrast, at the end of the colitis induction period, the colonic wall became thick and the epithelial architecture was destroyed. Under microscopy, distortion of crypts, loss of goblet cells, submucosal edema and linfoplasmacytic infiltration, with massive presence of mono- and polymorphonuclear cells, deep ulcerations, were observed, with cryptic abscesses (Figure 5B upper panel) in CD mice.

In Ph pretreated mice a lower evidence of cryptitis and linfoplasmacytic infiltrations was observed, with follicular like aspects and lesser neutrophils infiltration (Figure 5B lower panel). In the recovery phase, after 14 days DSS suspension, a linfoplasmacytic and neutrophils infiltrate at the base of the crypts, a sign of regenerative processes were found (Figure 5C upper panel) [53].

In Ph treated mice, minimal chronic linfoplasmacytic infiltrate was present with incipient and in some cases complete healing (Figure 5C lower panel).

### Plasma Lipids and Glucose

Total cholesterol, HDL- cholesterol, triglycerides and glucose before and at the end of the study (14 days after DSS colitis induction) in control mice and in mice fed phytosterols enriched diet are reported in Table 2. Phytosterols are effective in reducing plasma cholesterol levels. The anti-inflammatory effect of phytosterols has been therefore investigated at the same dose exerting the hypocholesterolemic activity.

**Table 2:** Plasma lipid and glucose parameters evaluated during the study.

	Cholesterol*	HDL-Cholesterol*	Triglycerides*	Glucose*
**Before the study**	88.3±3.2	60.2±1.3	160.5±6.2	206.0±13.4
**Day 0 (A)**				
**CD prevention**	89.3±3.7	59.2±1.3	159.5±6.3	206.7±15.0
**Day 14 (B)**				
**Ph prevention**	66.8±2.6	61.7±2.6	158.7±1.8	200.5±2.2
**Day 14 (C)**				
**CDinduction**	96±2.9	59.7±3.0	160.5±7.5	230.0±9.0
**Day 28 (D)**				
**Ph induction**	66.3±1.8	60.0±3.7	150.0±7.2	235±14.4
**Day 28 (E)**				
**CD remission**	97.2±2.7	58.3±3.2	162.5±7.9	205.0±8.0
**Day 38 (F)**				
**Ph remission**	67.3±0.8	62.0±3.7	159.0±7.2	198.0±15.4
**Day 38 (G)**				

*mg/100 ml. Cholesterol: A, B *vs* C: *P*<.01; A,B *vs* D *P*<.05. A, B *vs* E: *P*<.01.; A, B *vs* D *P*<.05. A,B *vs* G: *P*<.01 (mean ± SD, n = 3–6 animals). Statistical analysis was performed by using a two-tailed unpaired Student's *t* test for continuous data.

### Plasma, liver and biliary bile acids

**Figure 6:**
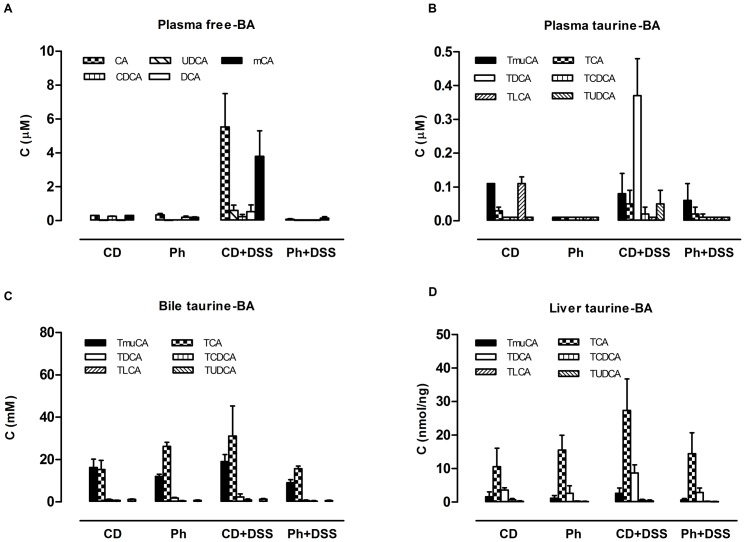
Plasma, liver and biliary BA. A. Plasma free and taurine-conjugated BA. B. Biliary BA. C. Liver BA. BA were evaluated after 14 days either Ph or CD and at the end of the study (day38). Since the CD group from day 0 to day 14 went on assuming the same usual commercial diet as before, day 14 was considered as the control value with respect to Ph. Plasma, liver and bile were collected from the same animal. The results were expressed as mean ± SD (n = 3–4 animals) CA: cholic acid; CDCA: chenodeoxycholic acid; DCA: deoxycholic acid; LCA: lithocholic acid; UDCA: ursodeoxycholic acid; muCA: muricholic acid; T-: Taurine conjugated-.

Plasma, liver and biliary bile acids were evaluated after 14 days of either CD or Ph at the end of the study. Data are reported in Figure 6 and in Tables S7A-C (detailed information is presented in File S7: Additional Results). Since the CD group from day 0 to day 14 assumedthe same usual commercial diet as before, day 14 was considered as the control value with respect to Ph.

Plasma: unconjugated BA predominated in both groups, T-conjugated BA accounted for a small fraction of plasma BA. The small amount of Taurine conjugated BA in serum is dependent on the intestinal input of bile acids deconjugated by intestinal bacteria. In CD and Ph fed animals plasma bile acids were very low; Ph induced a decrease in CDCA; after induction of DSS colitis, in CD fed mice, there was a significant increase in plasma BA, mainly unconjugated muCA, DCA and UDCA. In Ph fed mice, after induction of colitis, plasma bile acids were not increased with respect to Ph prevention period and DCA almost disappeared in plasma. Total BA were significantly lower than in CD group (Figure 6A and B).

In the liver of Ph fed mice, the BA pattern (Figure 6C) and concentration were similar in Ph mice, both before and after DSS, with values similar to those found in CD fed mice before colitis induction, with TCA being the predominant BA. After DSS, an increase in BA liver concentration was observed in the CD group, accounted for mainly by TCA and TDCA, the product of 7α intestinal bacteria deconjugation.

In bile, TCA predominated in Ph mice while TmuCA and TCA were the main BA in CD mice; after DSS, TCA increased in CD group, with an opposite trend in the Ph group (Figure 6D). In particular, TDCA increased in the CD group after DSS, but decreased in the Ph group.

The results demonstrate that DSS induced colitis is associated with an increase of secondary bile acids, due to the increased bacterial 7α-dehydroxylation, resulting from inflammation, which can be partly prevented by phytosterols.

### Functional *in vitro* studies

#### Gallbladder

**Figure 7:**
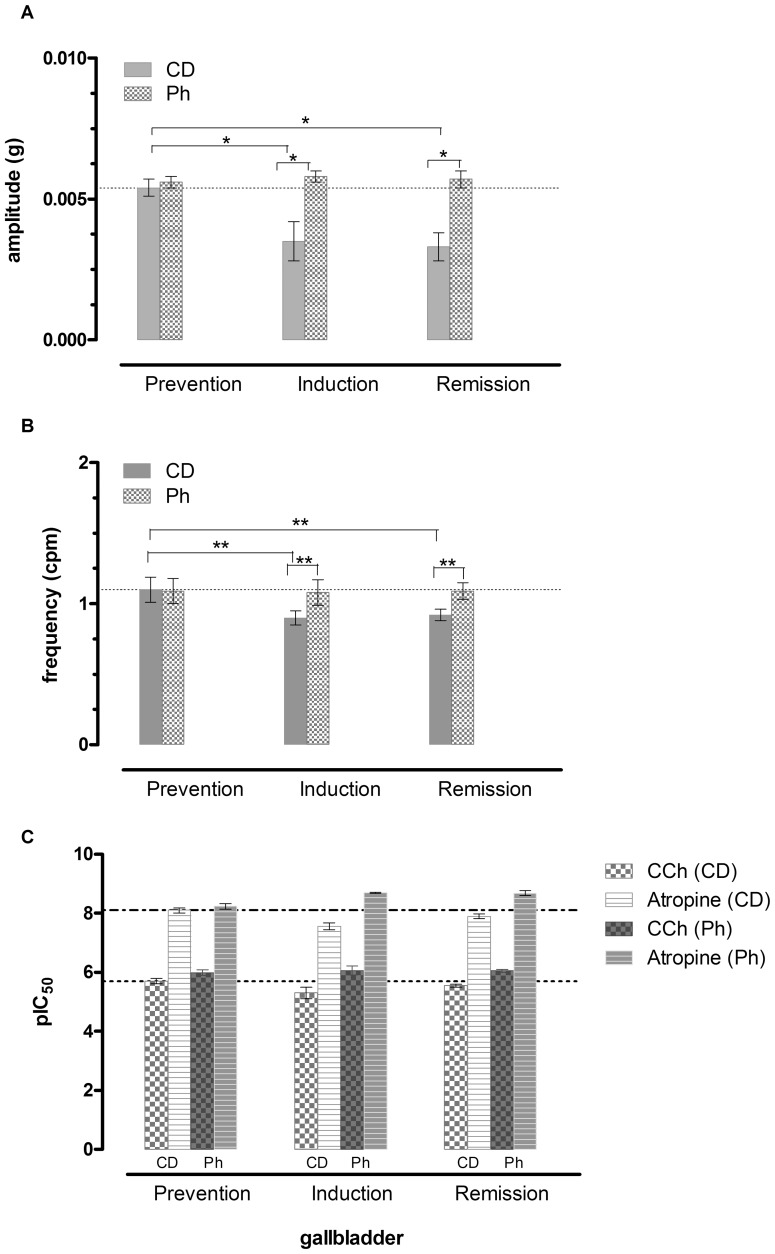
Gallbladder Spontaneous and Induced motility. Amplitude (A) and Frequency (B) of spontaneous phasic contractions and (C) response to CCh and Atropine in mice gallbladder. Data are mean ± SEM values (*n* = 3–5) (*P>.01) (**P>.05).

As we previously observed [49], spontaneous contractile activity of mouse isolated gallbladder smooth muscle was not regular: some strips only possessed tonic state, while others showed spontaneous phasic contractions. DSS induced colitis reduced both the amplitude and the frequency of spontaneous phasic contractions; gallbladder motility was not restored after DSS suspension. In phytosterols fed mice spontaneous phasic contractions were not modified throughout the study period and DSS induced motility alterations have not occurred. Amplitude and Frequency of the spontaneous basal motility are reported in Figures 7A. The representative traces of spontaneous phasic contraction are reported in Figure S7A in File S7.

The response to CCh and atropine remained constant in the colitis induction period,while it was increased in the remission in the Ph group. DSS reduced the response to CCh and increased the response to Atropine in the CD group and DSS suspension did not restore the gallbladder motility to the previous control conditions in CD mice (Figure S7A and Table S7D and S7E in File S7.).

#### Ileum

In control diet fed mice, ileal spontaneous contractions in the ileum were constant in amplitude and frequency. Ph did not modify amplitude and frequency of phasic basal ileal contractions. In control mice treated with DSS,partly organized waves characterized by decreased amplitude and increased frequency were observed. DSS treatment in Ph fed mice resulted in a slight decrease in frequency, with a constant amplitude of phasic contractions. DSS suspension did not restore the motility pattern in control diet fed mice, while Ph almost restored the primitive pattern. Therefore Ph seems to protect against the DSS induced disturbance in basal control pattern. Amplitude and Frequency of the spontaneous basal motility are reported in Figure 7B. The representative traces of spontaneous phasic contraction are reported in Figures S7B in File S7and Table S7C and D in File S7.

**Figure 8:**
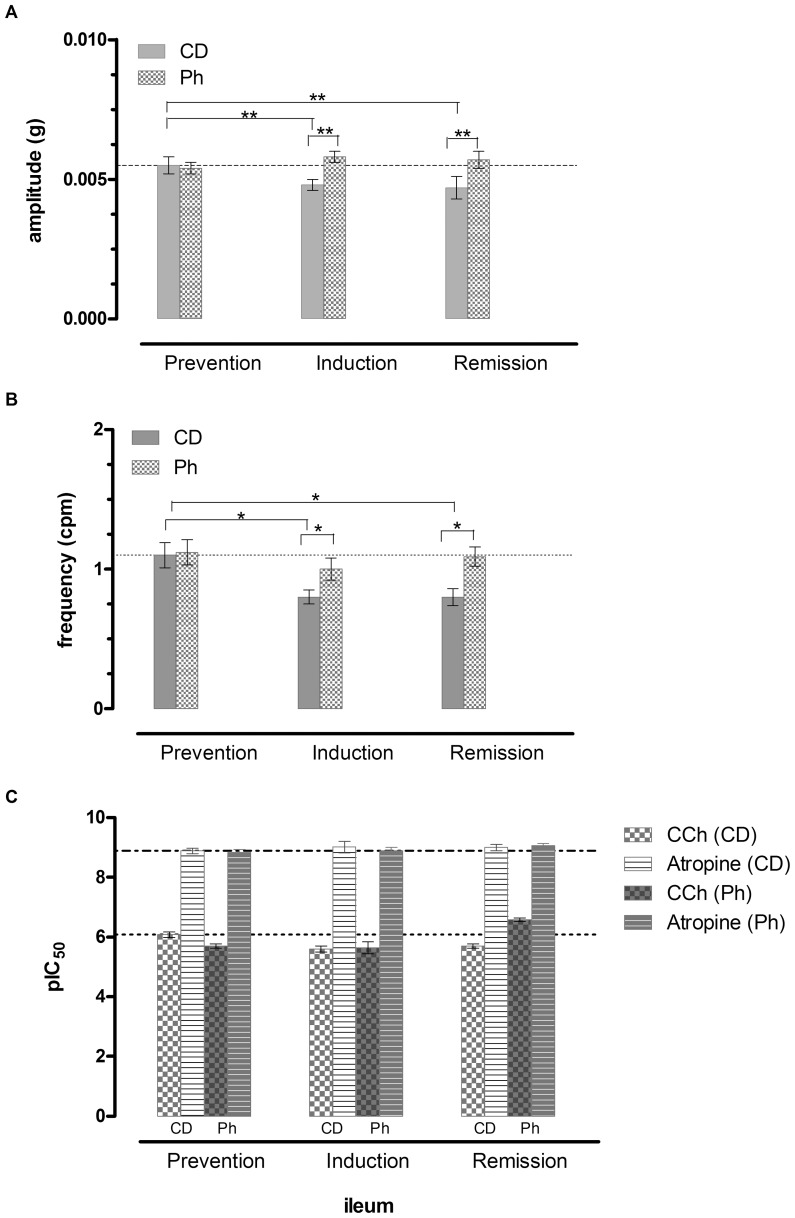
Ileum Spontaneous and Induced motility. Amplitude (A) and Frequency (B) of spontaneous phasic contractions and (C) response to CCh and Atropine in mice ileum. Data are mean ± SEM values (*n* = 3–5) (*P>.01) (**P>.05).

In the prevention period, Ph decreased the response to CCh, compared to CD fed mice and the response was unaffected by DSS. DSS reduced the response to CCh in CD mice. In the remission phase, in the CD group, the response to the cholinergic agent was decreased, while in the Ph group the response was increased, similarly to atropine response (Figure 8C).

#### Colon

**Figure 9:**
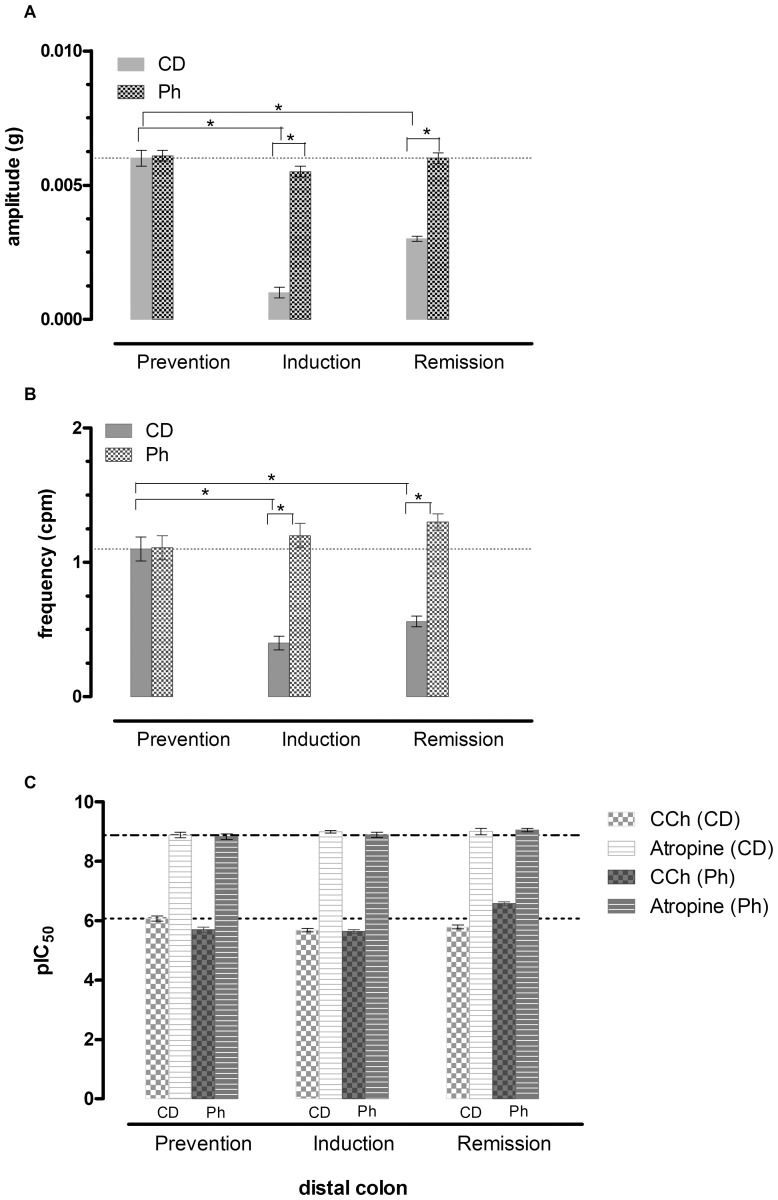
Colon Spontaneous and Induced motility. Amplitude (A) and Frequency (B) of spontaneous phasic contractions and (C) response to CCh and Atropine in mice colon. Data are mean ± SEM values (*n* = 3–5) (*P>.01) (**P>.05).

The colon of both CD and Ph groups presented similar phasic contractions of regular amplitude and frequency. DSS almost abolished the phasic contractions in the colonic strips, reducing both amplitude and frequency. After DSS suspension a partial recovery in amplitude, but not in frequency of the contractions was observed. Ph seem to protect against the DSS induced alteration: the phasic contractions were still present, though reduced in amplitude; DSS suspension resulted in a recovery of the amplitude and an increase in frequency with respect to the prevention period. Amplitude and Frequency of the spontaneous basal motility are reported in Figures 7C. The representative traces of spontaneous phasic contraction are reported in Figure S7C and Table S7C and D in File S7. DSS decreased the response to CCh with an associated increase in the response to Atropine with respect to the prevention period in CD fed mice. Therefore the induced motility was severely disturbed in acute colitis, and it was not recovered during the remission period. In addition, the response to CCh was decreased by Ph and it was not modified in the colitis induction period. On the contrary, the response was increased in the remission period. Similarly, the response to Atropine was increased (Figure 9C). The response was more evident than in the ileum.

## Discussion

In this study we have proven, for the first time, the effect of phytosterols on prevention, induction and remission of IBD using a murine model of acute colitis induced by DSS [54].

Recently, several studies have demonstrated that remission in patients with IBD should be defined not only by clinical improvement and by “normal appearance” of the intestinal mucosa at the endoscopy but also by achievement of mucosal healing [55].

Colonic inflammation was evaluated by histological findings and microPET due to the high correlation of microPET and histological score, demonstrated in adoptive transfer [56] and DSS induced colitis [37]. The comparison between histology and FDG microPET shows that increased neutrophil infiltration in the mucosa can be the cellular basis of the increased FDG signal in acute DSS-colitis. Of note, in the present investigation we have found a significant (*P*<.01) higher FDG uptake in the colon from Ph fed mice than in CD fed mice in the prevention period, consistently with a significantly greater metabolic activation of the organ. A low-grade activity is often found in the colon due to a combination of physiological factors, including normal intraluminal activity, i.e. bacterial metabolism, lymphoid tissue uptake, and smooth muscle uptake [52].

This low grade activation induced by Ph which we have showed exerting a protective effect against the DSS induced damage, and to regulate the intestinal microflora (see below) and mucosal activity can be one among the mechanisms underlying the beneficial effect of phytosterols in mice induced experimental colitis.

In both the remission and induction phase, there were significant differences between the Ph and CD group. The induction phase was associated with body weight loss, visible fecal blood, diarrhea, and other histopathological changes such as ulcerations, polymorphonuclear cells and MNC infiltration, loss of epithelial structures. In the remission phase (after DSS suspension), an improvement of clinical symptoms was associated to histopathological findings of reduction of the inflammatory infiltrate as well as reparation of epithelial structure in both groups, but significantly accelerated in Ph fed mice, where incipient healing was observed. Therefore, Ph pretreatment, if it does not prevent the onset of colitis, significantly decreases its clinical severity in the acute phase and reduces tissue inflammation and improves significantly mucosal healing duringremission. The colon of the DSS-treated mice showed complete destruction of the epithelial architecture with extensive depletion of goblet cells in the mucosa, disordered gland architecture loss of crypts, cryptic abscesses, submucosal edema, and intense inflammatory cellular infiltration in all layers. Pretreatment with phytosterols attenuated the morphologic damage and reduced the infiltration of leukocytes, in addition to decreasing mucosal disruption. In the remission phase, in the CD group a reduced inflammatory infiltrate was observed, while in the Ph group the reparation was almost complete with mucosal healing. Since Ph pretreatment reduces mucosal inflammation and the infiltration of leukocytes in the DSS-induced colitis, it is conceivable that another mechanism underlying the protective effects of phytosterols involves a reduced infiltration of inflammatory cells into the colonic mucosa both in the induction and in the remission phase. The accumulation of inflammatory cell infiltrates such as neutrophils contributes to reactive oxygen species production that triggers the destruction of tissues. It could be nice to speculate: leucocytes trafficking? Maybe through remodeling of the epithelium?

To evaluate the level of inflammation as index of severity of disease, we also determined the levels of reactive metabolites of oxygen and nitrogen. Inflammation causes an increase in the production of reactive oxygen species with damage by oxidizing proteins, lipids, nucleic acids, and other cellular components. In particular, the lipid peroxidation can alter membrane permeability and function. MDA levels, indicative of increased oxidative damage to lipids, were increased in the colon and ileum in mice in the induction phase, and decreased in the remission phase. MDA levels were significantly higher in the CD than in Ph group. Ph pretreatment, even if it does not completely prevent the oxidative stress associated to the acute phase of the disease, was effective in the reduction of the lipids oxidative damage. The lower MDA levels found in the intestinal tissues of Ph fed mice compared to the levels found in CD fed mice are consistent with the micro PET results and the clinical evaluation of the disease. Therefore, we suggest that Ph, together with the inflammatory condition, would simultaneously change the oxidative state in DSS-induced inflammation.

Therefore Ph exert their activity both through a stabilization of the membrane structure, thus preventing lipid peroxidation, and through their antioxidant properties, thereby conferring protection against DSS damage Recent observations indicate that phorbol esters-stimulated macrophages, from Ph fed rats, release lower amounts of the superoxide anion [57]. Many studies indicate that ordered lipid domains (rafts) composed of relatively saturated lipids (sphingolipids) and sterol play important roles in many biological processes. Moreover, it is known that phytosterols are poorly absorbed from food and that they are present in human membranes [58], either increasing total sterols concentration or, more possibly, replacing cholesterol [59]. So far, there are not conclusive data whether incorporation of phytosterols in membranes influence their properties and alter their function [60] [61]. It is known that oxidative stress may induce a reduction in Coenzyme Q levels that, beyond its central role in bioenergetic metabolism, is also a powerful antioxidant. For this reason we have measured the CoQ_9_ (the predominant form in rodents) tissue concentration in colon specimens. CoQ_9_ levels appeared to be unaffected by acute colitis induction both in presence or in absence of phytosterols pre-treatment. However, the slight tendency to increased levels of CoQ9 in inflamed tissues can be due to a compensatory mechanisms allowing its recruitment from circulating lipoproteins. In conclusion, the oxidative stress can decrease the tissue levels of Co-enzyme C9, but the increased blood supply in the inflamed tissue can provide further Co-enzyme C9, balancing the decrease.

Since animal models of colitis have shown defects in motility, we wondered whether phytosterols exert their protective effect also towards intestinal motility alterations occurring in IBD. Interestingly, our study revealed that DSS induces a disturbance in basal and stimulated gallbladder motility that is not completely normalized by DSS suspension. phytosterols exert a protective effect on gallbladder spontaneous and CCh induced motility. Since phytosterols cannot exert a direct effect on the gallbladder muscular layers, this finding is intriguing.

While gallbladder emptying has long been known to be stimulated by cholecystokinin (CCK), released from the proximal duodenum in response to feeding, gallbladder filling regulation is poorly understood. Quite recently, Intestinal Human Fibroblast Growth Factor (FGF)19 and its mouse orthologue FGF15, released from the ileum after a meal [62], as a result of ileal Farnesoid X Receptor (FXR) activation has been shown to oppose CCK effects. Besides FGF15(19), TGR5 expressed in muscular gallbladder cells [63] causes gallbladder smooth muscle relaxation, independently of CCK [64]. Since both FXR [65] and TGR5 [66] are strongly activated by DCA, it is possible that decreased gallbladder motility in DSS treated mice is due to the increase in DCA.

Before DSS treatment, in the Ph group, plasma DCA levels, the product of 7α-deconjugation of CA by intestinal bacteria, were higher than in the CD group, consistent with an increase of intestinal deconjugating bacteria induced by phytosterols. This latter finding is in agreement with the higher^18^F-FDG uptake in the Ph than in the CD group before DSS administration (see above).

After DSS, in CD fed mice, DCA increased compared with the Ph group, showing that DSS is associated with a severe bacterial colonization, partly prevented by phytosterols, which can exert their protective activity through a regulatory effect of the intestinal microbiota. It is well-known that colonic contractility is reduced in IBD [67] and the disturbance is persistent after the resolution of inflammation [68]. The decreased motor function is due both to receptor-dependent and receptor-independent mechanisms. We have shown [35] in a previous study and in the present investigation that impaired spontaneous and induced motor function is present also in the non inflamed ileum. Since the enteric nervous system is deranged in patients with IBD, with abnormalities of different degree, it has been postulated [69] that these abnormalities may impair the neurophysiological architecture. In TNBS guinea pig ileitis, ileal inflammation alters the enteric reflex circuits in non-inflamed regions of the intestine [70] and these modifications are considered responsible for the altered function in non-involved segments during episodes of intestinal inflammation. DSS colitis represents a different model from TNBS ileitis, but similarly to the TNBS model, the motor disturbances are present also in non inflamed intestinal segment both in the spontaneous phasic activity and in the stimulated muscular contractions. This study has determined that phytosterols prevents alterations on basal phasic contractions in the ileum in both the acute and remission phase. In the colon, DSS colitis almost abolishes the spontaneous phasic contractions. phytosterols, reducing inflammatory cells infiltration and decreasing oxydative stress, protect from colonic muscle relaxation.

The latter finding is of relevance since small bowel distension can be a predictor of toxic megacolon and, when persistent, it has been reported to be a risk of possibly multiorgan dysfunction (‘impending’ megacolon) [71]. In the acute phase of colitis, the response of the ileal and colonic muscle strips to the cholinergic agent carbachol is decreased both in CD fed mice and in Ph mice. During reparation, the response to carbachol is decreased in CD fed mice in both the intestinal segments, but it is surprisingly increased in both the tracts in Ph fed mice. The increase of the response both to CCh and atropine highly supports the thesis of a rise in amount of the number (up regulation) of Cholinergic receptors on the muscle membrane. Muscarinic receptors are present throughout the gut in the muscle [72] and epithelial cells [73] and their activity is dependent on the integrity of the cell membrane and it is impaired by intestinal infection and inflammation. Numerous studies have shown that intestinal infection and inflammation impair the muscarinic cholinergic response of the gut epithelium [73].

A complex network of factors regulates healing of the epithelium and the restitution of epithelial barrier function among which the activation of M1 muscarinic acetylcholine receptor (mAChR) both in IBD patients [74] and in mouse model of colitis [75]. A reduced number of M1mAChR has been found in inflamed areas and evidence has been produced that the reduction of mAChRs can be a fundamental mechanism of inflammatory cholinergic hyporesponsiveness. In the present work we have shown that phytosterols restore the responsiveness of the inflamed intestine to CCh and atropine. Since atropine is a potent non selective muscarinic receptor antagonist not targeting M1 mAChR only, the increaed response to atropine can be due to an increased number of atropine receptors. This finding supports the concept that phytosterols can also act at the level of the cholinergic system.

## Conclusions

Although phytosterols anti-inflammatory properties are well known and already described [12] [13] [14] [15] [16], the absence of information on proinflammatory cytokines and mediators in blood and intestinal tissue is a major limitation of this study, since different sterols and a different dosage were employed. Further limitation of the present investigation is the murine model and the non reliability of these results for humans, the lack of a confirm of phytosterols effect in a spontaneous setting rather than the induced colitis, and the very small sample size. Despite these drawbacks, our study shows for the first time that phytosterols pretreatment reduces the clinical symptoms and exerts a protective effect on DSS induced colonic inflammation decreasing infiltration of inflammatory cells and accelerating mucosal healing. These effects can be related to their antioxidant effects and to a regulation of the intestinal microflora, as indirectly shown by the modification in the bile acid pattern.phytosterols play also a role in the restoration of the intestinal motor pattern. These findings pavethe way towards the role of phytosterols as potential nutraceutical tools in the management of IBD and other intestinal inflammatory diseases.

## Supporting Information

File S1 **About Phytosteols Preparation.**
(DOC)

File S2 **Phytosterols Administration.**
(DOC)

File S3 **DSS Induction of Colitis.**
(DOC)

File S4 **Assessment of Inflammation.**
(DOC)

File S5 **Determination of Plasma, Liver and Biliary Bile Acids.**
(DOC)

File S6 **Chemicals.**
(DOC)

File S7 **Additional Results.**
(DOC)

File S8 **Supporting Additional References.**
(DOC)
