# Supplementary material for: Antiinflammatory Effect of Phytosterols in Experimental Murine Colitis Model: Prevention, Induction, Remission Study
Source: PLoS One. 2014 Sep 30;9(9):e108112. doi: 10.1371/journal.pone.0108112 (PMC4182327; doi:10.1371/journal.pone.0108112)
Supplement: File S1 — About Phytosteols Preparation. (DOC) [file pone.0108112.s001.doc]

**S1. About phytosterols preparation**

Administered phytosterols were supplied by Solgar (Solgar Italia® Multinutrient® S.p.A. Via Prima Strada, 23 int. 3 - 35129 Padova – Italy). The detailed composition of the nutraceutical preparation is reported in Table S1. Further information about phytosterols are available at Solgar website (<http://www.solgar.it/>).

**Table S1.** Composition of Phytosterols preparartion

| Ingredient |  | Quantity (mg/4 pearls) |
| --- | --- | --- |
| Mixture of phytosterols (from soy)*^1^* |  | 2000 mg |
|  | Total plant sterol esters | 1800 mg |
| edible gelatin |  | 931 mg |
| Agent of resistance |  |  |
|  | Glycerol | 429 mg |
| Safflower (*cartamus tintorius*) oil*^2^* |  | 180 mg^1^ |
| Dye | Caramel | 68 mg |

*^1^*Phytosterols (plant sterols) are members of the ‘‘triterpene’’ family of natural products, which includes more than 100 different phytosterols and more than 4000 other types of triterpenes. These molecules are similar to cholesterol (Figure S1), both in structure, given the four-ring steroid nucleus, the 3β-hydroxyl group and often a 5,6-double bond and they can be divided them into three groups based on the number of methyl groups on carbon-4, two (4-dimethyl), one (4-monomethyl), or none (4-desmethyl). 4-Dimethylsterols and 4a-monomethylsterols are metabolic intermediates in the biosynthetic pathway leading to end-product, 4-desmethyl phytosterols, but they are usually present at low levels in most plant tissues [RS1]. In soy, the most common phytosterols include β-sitosterol, stigmasterol, campesterol, in addition to campestanol, sitostanol (Figure S1) [RS2].

*^2^Carthamus tintorius* L. oil is used, in Folk Medicine, as coadjuvant in the treatment of various diseases such as rheumatism, sores, and other inflammatory diseases involving the gastrointestinal tract. Its chemical composition includes, among others, phytosterols, fatty acids [RS3].

**Figure S1.** Chemical structure of the main administered phytosterols in comparison with cholesterol.
