# Supplementary material for: Antiinflammatory Effect of Phytosterols in Experimental Murine Colitis Model: Prevention, Induction, Remission Study
Source: PLoS One. 2014 Sep 30;9(9):e108112. doi: 10.1371/journal.pone.0108112 (PMC4182327; doi:10.1371/journal.pone.0108112)
Supplement: File S2 — Phytosterols Administration. (DOC) [file pone.0108112.s002.doc]

**S2. Phytosterols ADMINISTRATION**

The calculation of the human equivalent amount of phytosterols consumed by mice has been based on the following assumptions: the recommended dose for human is 1.8 g/day and the average adult human weighs 65 kg. This accounts for a dose of 27.6 mg/kg for man. Our calculation of the human equivalent amount of phytosterols consumed by mice uses the body surface area normalization method (translational dose) with the following assumptions: a mouse eats 5g chow daily and weighs 22-25 g; chow contains 2g/kg of phytosterols. Therefore, 2/1000g chow per 5 g chow/day gives 0.01 g daily. If a mouse weighs an average 25 g, then 10 mg/25 g per1000 gives 400 mg/kg daily. As discussed by Reagan-Shaw [RS4], the human equivalent dose (mg/kg) is equal to animal dose (mg/kg) per(animal Km/human Km). As such, human equivalent dose (mg/kg) for mouse is 400 mg/kg per (3/37) consistent with the recommended dose for human of 1.8 g/ day.

Phytosterols are thought not to be absorbed by the intestine, but we have considered it correct to follow these indications due to the complex interplay of multiple processes, among which the so called transintestinal cholesterol excretion [RS5] and competition with cholesterol for absorption and in absence of other guide lines.
