# Supplementary material for: Antiinflammatory Effect of Phytosterols in Experimental Murine Colitis Model: Prevention, Induction, Remission Study
Source: PLoS One. 2014 Sep 30;9(9):e108112. doi: 10.1371/journal.pone.0108112 (PMC4182327; doi:10.1371/journal.pone.0108112)
Supplement: File S3 — DSS Induction of Colitis. (DOC) [file pone.0108112.s003.doc]

**S3. Induction of DSS Colitis**

Acute colitis were induced by DSS administration according to Waldner [RS6] and Wirtz [RS7] After 14 days of either phytosterols (Ph) or control diet (CD) assumption, mice were weighted and marked in their tail. DSS (5% w/v) was added to the drinking water: The DSS amount consumed was measured daily: 5 ml DSS solution per mouse per day was considered the average amount that a mouse must drink to introduce a sufficient amount of DSS. Whenever it did not occur, the animal was excluded. The DSS solution was changed every third day up to the tenth day when it was replaced by normal water. Every other day mice were weighted. Stools were evaluated for consistency and presence of blood.

*Experimental Procedure* Twenty four hours before the experiments, food was withdrawn and water was maintained *ad libitum*. The animals were sacrificed by cervical dislocation. Immediately before the sacrifice, blood was withdrawn by cardiac puncture in heparinized tubes and plasma was frozen at -80°C for BA analysis. The abdomen was opened by median laparotomy and exposed. The gallbladder was visualized, the cystic duct tied and dissected from the common bile duct. Bile was removed with an insulin syringe (29G) and stored at -80°C until BA analysis. The removed gallbladder was immediately used for motility studies.

A portion of jejunum, immediately after the ligament of Treitz was removed (1.5 cm) and retained for histological examination. A 1.5 cm length segment of the terminal ileum, immediately proximal to the ileo-cecal valve and a 2.5 cm region of the distal colon were identified, gently flushed with Krebs solution to remove fecal residues and dissected into two parts: one part was placed in 10% formalin for subsequent histological analysis, a second was retained for immediate in vitro motility studies (see below) or frozen at -80°C for oxydative stress analysis. A liver sample (2g) was collected and frozen at -80°C for BA analysis.

Before the study and after the study was completed, total cholesterol, HDL Cholesterol, triglycerides and glucose were measured on plasma samples taken by cardiac puncture respectively from 6 control animals, 6 animals receiving phytosterols and 6 animals receiving control diet, 14 days after DSS was stopped and each group had continued its own diet. triglyceride, HDL-Cholesterol, total cholesterol and glucose concentrations were measured using Dimension RxL Max system (Siemens Healthcare Diagnostics, Newark, DE, USA), following manufacturer’s instructions. For each mouse a plasma specimen volume of 100 µl was tested.
