# Supplementary material for: Antiinflammatory Effect of Phytosterols in Experimental Murine Colitis Model: Prevention, Induction, Remission Study
Source: PLoS One. 2014 Sep 30;9(9):e108112. doi: 10.1371/journal.pone.0108112 (PMC4182327; doi:10.1371/journal.pone.0108112)
Supplement: File S4 — Assessment of Inflammation. (DOC) [file pone.0108112.s004.doc]

**S4. Assessment of inflammation**

*Symptoms and colon length.*

Clinical assessment of inflammation included daily monitoring of body weight and general health conditions. At necropsy, the macroscopic appearance of the colon, the presence visible fecal blood, blood in peritoneum and ulcerations were recorded. The colon was cut close to the ileocecal valve and rectum, and the length was measured.

*Histology and histological score.*

Five micron sections were cut and stained with Haematoxylin and Eosin (H&E). The histological preparations were examined histologically on a blinded basis. The histological grading was evaluated as follows. Acute colitis is characterised by intense inflammatory cell infiltrate, crypt abscesses, mucin depletion, and surface ulceration, that are the main histological features of acute activity. The hallmark is the presence of neutrophils and eosinophils infiltrating *lamina propria* and crypt epithelium. Therefore, according to the amount of acute inflammatory cell infiltrate in colon and colon and in small bowel slides colitis was classified in four grades as reported in Table S4.

**Table S4.** Histological score for the evaluation of the inflammation in ileal and colonic tissues from mice throughout the study.

| **0** | No inflammatory infiltration of neutrophyls and eosinophils in the lamina propria. |
| --- | --- |
| **1** | Mild and focal infiltrate of neutrophils and eosinophils in the lamina propria with mild crypt aggression. |
| **2** | Moderate infiltrate of neutrophils and eosinophils in the lamina propria with moderate crypt aggression. |
| **3** | Marked and diffuse infiltrate of neutrophils and eosinophils in the lamina propria with marked crypt aggression. |
