# Supplementary material for: Antiinflammatory Effect of Phytosterols in Experimental Murine Colitis Model: Prevention, Induction, Remission Study
Source: PLoS One. 2014 Sep 30;9(9):e108112. doi: 10.1371/journal.pone.0108112 (PMC4182327; doi:10.1371/journal.pone.0108112)
Supplement: File S5 — Determination of Plasma, Liver and Biliary Bile Acids. (DOC) [file pone.0108112.s005.doc]

**S5. Determination of Plasma, Liver and Biliary Bile Acids**

Total bile acids in plasma, liver and bile have been determined by HPLC-ES-MS/MS.[RS9] Briefly HPLC was performed using a 2695 Alliance system (Waters, Milford, MA) separation module coupled with autosampler. The analytical column was a Waters XSelect CSH Phenyl-Hexyl (150x2.0mm i.d., 4 µm particle size), protected by a Security Guard ODS 4 x 2.0 mm i.d. guard column, both supplied from Waters.

The sample preparation (extraction method) is different from a matrix to another.

-*Plasma samples*: plasma samples (100 µl) were diluted 1:6 (v/v) with NaOH 0.1N and heated to 64°C for 30 minutes. The solid phase extraction (SPE) cartridge was conditioned with 5 ml methanol and 5 ml of water prior to sample loading. Plasma samples were loaded into the conditioned cartridge and then washed with 10 ml water. The cartridge was then eluted with 5 ml methanol and the eluate was collected, then dried under vacuum and reconstituted with 100 µl mobile phase and 5 µl were injected into the HPLC-ESI-MS/MS instrument.

-Plasma free (for calibration curve): charcoal was rinsed several times, then dried. 50 mg of charcoal was put in every 1 ml of plasma and shaken. After stirring at +4 °C overnight, the plasma was centrifuged at 3000 rpm for 5 minutes, then filtered through Millipore 0.22 µm and stored at -20 ° C.

-*Liver samples*: each mouse liver sample was weighed and 0.20 g ± 0.02 g was taken from different points of the sample; 1 ml phosphate buffer (0.005 M, pH 7.2) was added and the mixture was homogenized ,washed with methanol (1.5 ml). The mixture was sonicated for 5 minutes, vortexed for 2 minutes, heated to 37ºC for 20 minutes, and centrifuged at 4000 rpm for 15 minutes. 200 µl of the supernatant was dried under vacuum and resuspended with 2 ml sodium hydroxide (0.1 N). The solution was sonicated for 10 minutes, heated to 64ºC for 30 minutes, and SPE was carried out on C18 extraction cartridges. The SPE cartridge was conditioned with 5 ml methanol and 5 ml water prior to sample loading. The liver sample extract was loaded onto the conditioned cartridge and washed with 10 ml water. The cartridge was then eluted with 5 ml of methanol. The eluate was dried under vacuum and reconstituted with 100 µl mobile phase solution; 5 µl were injected into the HPLC-ESI- MS/MS instrument.

-*Bile samples*: mouse bile samples were brought to room temperature, briefly stirred, and 1:100 (v/v) with mobile phase. Final solution was transferred in autosampler vials, and 5 µl were injected into the HPLC-ESI- MS/MS instrument.

Bile acids from different media were separated in elution gradient mode using 15 mM ammonium acetate buffer (pH = 8.00) as mobile phase A and acetonitrile: methanol =75:25 (v/v) as mobile phase B. Mobile phase B was increased from 30% to 45% in 10 min, then to 70% in 10 min, and finally brought to 100% in 1 min and held constant for 3 min. Injected sample volume was 5 µL. Flow rate was 150 µL/min and the column was maintained at 45 °C. The HPLC is combined with a triple quadrupole mass spectrometer with an Electrospray source operating in negative ionization (HPLC-ESI-MS/MS) using a Quattro-LC (Micromass) triple quadruple mass spectrometer operating in Multiple Reaction Monitoring (MRM) acquisition mode. MassLynx software version 4.0 was used for data acquisition and processing. Nitrogen was used as nebulizer gas at 100L/hr flow rate and as desolvation gas at 610L/hr. Ion source block and desolvation temperatures were set at 120 °C and 180 °C, respectively. Capillary voltage was 2.5 kV and cone voltage was 60 V. Full-scan mass spectra and product ion spectra of each bile acids are obtained in the optimized mass spectrometry conditions. Chromatograms were acquired using mass spectrometer in multiple reaction monitoring (MRM) mode Collision energies was performed for each bile acids. The Micromass Mass-Lynx version 4.0 software was employed for instrument control, data acquisition, and processing. Calibration samples were obtained in the 0.05 to 10 μmol/L concentration range prepared in plasma free (same preparation used for the samples) and only in mobile phase. Linear calibration curve parameters were obtained from the plot of the analyte peak area versus Internal standards concentration using a least squares regression analysis (weight = 1/x). Correlation coefficients were higher than 0.991).
