# Supplementary material for: Antiinflammatory Effect of Phytosterols in Experimental Murine Colitis Model: Prevention, Induction, Remission Study
Source: PLoS One. 2014 Sep 30;9(9):e108112. doi: 10.1371/journal.pone.0108112 (PMC4182327; doi:10.1371/journal.pone.0108112)
Supplement: File S6 — Chemicals. (DOC) [file pone.0108112.s006.doc]

**S6.** **Chemicals**

Phytosterols were obtained from Solgar (Solgar, Solgar Italia® Multinutrient® S.p.A. Via Prima Strada, 23 int. 3 - 35129 Padova – Italy)].

DSS (m.w. 36.000–50.000) was obtained from MP Biomedicals (MP Biomedicals, Solon; OH, USA. All solvents were of high purity and used without further purification. All solvents were purchased from: Water LiChrosolv® for HPLC, Merck; Acetonitrile, LiChrosolv® for HPLC, Merck; Methyl alcohol RPE, Ammonia solution 30% RPE, Glacial acetic acid RPE, Carlo Erba Reagent; activated charcoal from Sigma Aldrich. C18 SPE columns ISOLUTE 500 mg 6 mL, for the plasma sample pretreatment, were purchased from StepBio (Bologna, IT). The standards of the different endogenous bile acids were obtained from Sigma Aldrich (St. Louis, USA); 6α-Ethil-chenodeoxicolic acid (6-ECDCA) and its tauro-conjugate were supplied by Prof. Roberto Pellicciari, University of Perugia, Italy and were highly pure (> 99 %) as documented.
