# Supplementary material for: Antiinflammatory Effect of Phytosterols in Experimental Murine Colitis Model: Prevention, Induction, Remission Study
Source: PLoS One. 2014 Sep 30;9(9):e108112. doi: 10.1371/journal.pone.0108112 (PMC4182327; doi:10.1371/journal.pone.0108112)
Supplement: File S7 — Additional Results. (DOC) [file pone.0108112.s007.doc]

**S7. Additional Results**

**Table S7A. Plasma bile acids concentration**

| **plasma BA**  **(μM)** | **CD** | **Ph** | **CD+DSS** | **Ph+DSS** |
| --- | --- | --- | --- | --- |
| **CA** | 0.30 ± 0.01 | 0.34 ± 0.09 | 5.5 ± 0.1 | 0.07 ± 0.04 |
| **UDCA** | 0.01 ± 0.00 | 0.01 ± 0.00 | 0.6 ± 0.3 | 0.04 ± 0.01 |
| **CDCA** | 0.25 ± 0.01 | 0.03 ± 0.01 | 0.2 ± 0.1 | 0.02 ± 0.02 |
| **DCA** | 0.01 ± 0.00 | 0.20 ± 0.06 | 0.5 ± 0.2 | 0.02 ± 0.01 |
| **muCA** | 0.30 ± 0.01 | 0.19 ± 0.03 | 3.8 ± 1.5 | 0.14 ± 0.09 |
| **TmuCA** | 0.11 ± 0.01 | 0.01 ± 0.00 | 0.08 ± 0.06 | 0.06 ± 0.05 |
| **TCA** | 0.03 ± 0.01 | 0.01 ± 0.00 | 0.05 ± 0.04 | 0.020 ± 0.005 |
| **TDCA** | 0.01 ± 0.00 | 0.01 ± 0.00 | 0.4 ± 0.1 | 0.010 ± 0.005 |
| **TCDCA** | 0.01 ± 0.00 | 0.01 ± 0.00 | 0.020 ± 0.005 | 0.01 ± 0.00 |
| **TLCA** | 0.11 ± 0.02 | 0.01 ± 0.00 | 0.01 ± 0.00 | 0.01 ± 0.00 |
| **TUDCA** | 0.01 ± 0.00 | 0.01 ± 0.00 | 0.05 ± 0.04 | 0.01 ± 0.00 |

CA: CD+DSS vs Ph+DSS *P* < .05; UDCA: CD+DSS vs Ph+DSS *P* < .05; CDCA: CD+DSS vs Ph+DSS and CD vs Ph *P* < .05; DCA: CD+DSS vs Ph+DSS and CD vs Ph *P* < .05; muCA: CD+DSS vs Ph+DSS *P* < .05; TmuCA: CD vs Ph *P* < .05; TLCA: CD vs Ph *P* < .05.

**Table S7B.** Liver bile acids concentration

| **liver BA**  **(nmol/g)** | **CD** | **Ph** | **CD+DSS** | **Ph+DSS** |
| --- | --- | --- | --- | --- |
| **TmuCA** | 2 ± 1 | 2 ± 1 | 3 ± 2 | 0.6 ± 0.4 |
| **TCA** | 10 ± 6 | 16 ± 4 | 27 ± 9 | 14 ± 6 |
| **TDCA** | 4 ± 1 | 3 ± 2 | 9 ± 2 | 3 ± 1 |
| **TCDCA** | 0.7 ± 0.4 | 0.2 ± 0.1 | 0.6 ± 0.3 | 0.2 ± 0.1 |
| **TLCA** | 0.02 ± 0.01 | 0.01 ± 0.00 | 0.03 ± 0.01 | 0.01 ± 0.00 |
| **TUDCA** | 0.20 ± 0.05 | 0.10 ± 0.03 | 0.4 ± 0.3 | 0.1 ± 0.08 |

TmuCA: CD+DSS vs Ph+DSS *P* < .05; TCA: CD+DSS vs Ph+DSS *P* < .05; TDCA: CD+DSS vs Ph+DSS *P* < .05; TCDCA: CD+DSS vs Ph+DSS *P* < .05; TUCA: CD+DSS vs Ph+DSS *P* < .05; TLCA: CD+DSS vs Ph+DSS *P* < .05.

**Table S7C.** Biliary bile acids concentration

| **bile BA**  **(mM)** | **CD** | **Ph** | **CD+DSS** | **Ph+DSS** |
| --- | --- | --- | --- | --- |
| **TmuCA** | 16 ± 4 | 12 ± 1 | 19 ± 3 | 9 ± 1 |
| **TCA** | 15 ± 4 | 26 ± 2 | 31 ± 14 | 16 ± 1 |
| **TDCA** | 1.0 ± 0.3 | 1.9 ± 0.2 | 2 ± 1 | 0.5 ± 0.4 |
| **TCDCA** | 0.7 ± 0.1 | 0.4 ± 0.1 | 0.9 ± 0.4 | 0.3 ± 0.1 |
| **TLCA** | 0.010 ± 0.005 | 0.002 ± 0.001 | 0.02 ± 0.01 | 0.004 ± 0.001 |
| **TUDCA** | 1.2 ± 0.2 | 0.7 ± 0.1 | 1.3 ± 0.3 | 0.6 ± 0.1 |

TCA: CD vs Ph *P* < .05; TLCA CD vs Ph *P* < .05.

Prevention

**CD Ph**


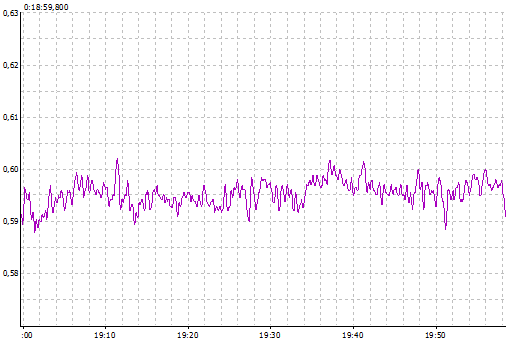


Induction

**CD Ph**

Remission

**CD Ph**

**Figure S7A.** Representative traces of the smooth muscle spontaneous motility in the Prevention, Induction and Remission period in CD (left) and in Ph (right) mice gallbladder. X axis (g) and Y axis (h). All the traces are taken from Power Lab files.

Prevention

**CD Ph**

Induction

**CD Ph**

Remission

**CD Ph**

**Figure S7B.** Representative traces of the smooth muscle spontaneous motility in the Prevention, Induction and Remission period in CD (left) and in Ph (right) mice ileum (*B*). X axis (g) and Y axis (h). All the traces are taken from Power Lab files.

Prevention

**CD Ph**

Induction

**CD Ph**

Remission

**CD Ph**

**Figure S7C.** Representative traces of the smooth muscle spontaneous motility in the Prevention, Induction and Remission period in CD (left) and in Ph (right) mice colon. X axis (g) and Y axis (h). All the traces are taken from Power Lab files.

**Table S7D.** Agonist (carbachol) and antagonist (atropine) affinities expressed as pEC_50_ or p*A*_2_ respectively on the isolated mice gallbladder, ileum and distal colon of control mice and phytosterols fed mice.

|  |  | **Prevention** | | **Induction** | | **Remission** | |
| --- | --- | --- | --- | --- | --- | --- | --- |
|  |  | **CD*^a^*** | **Phytosterols*^b^*** | **CD*^a^*** | **Phytosterols*^b^*** | **CD*^a^*** | **Phytosterols*^b^*** |
| **Gallbladder** | **CCh*^c^*** | 5.70 ± 0.09 | 5.99 ± 0.10 | 5.30 ± 0.20 | 6.06 ± 0.15 | 5.55 ± 0.05 | 5.85 ± 0.08 |
|  | **Atropine*^d^*** | 8.10 ± 0.09 | 8.23 ± 0.09 | 7.56 ± 0.11 | 8.70 ± 0.02 | 7.89 ± 0.08 | 8.68 ± 0.08 |
| **Ileum** | **CCh*^c^*** | 6.08 ± 0.09 | 5.70 ± 0.10 | 5.60 ± 0.10 | 5.65 ± 0.20 | 5.70 ± 0.07 | 6.58 ± 0.06 |
|  | **Atropine*^d^*** | 8.89 ± 0.09 | 8.84 ± 0.09 | 9.01 ± 0.20 | 8.90 ± 0.10 | 9.00 ± 0.11 | 9.21 ± 0.08 |
| **Distal colon** | **CCh*^c^*** | 6.08 ± 0.09 | 5.70 ± 0.10 | 5.69 ± 0.06 | 5.64 ± 0.06 | 5.80 ± 0.07 | 6.58 ± 0.06 |
|  | **Atropine*^d^*** | 8.89 ± 0.09 | 8.84 ± 0.09 | 9.00 ± 0.04 | 8.90 ± 0.09 | 9.01 ± 0.11 | 9.26 ± 0.06 |

*^a^* Tissues from healthy mice fed control diet. *^b^* Tissues from mice fed phytosterols. *^c^* Potency of CCh was expressed as pEC_50_. pEC_50_ = –log EC_50_. EC_50_ values are the means ± SE of at least four independent experiments and were calculated by a non linear regression curve-fitting computer program.[9] *^d^* Potency of competitive antagonism of atropine was expressed as p*A*_2_. p*A*_2_ values ± SE were calculated from Schild plots [10] constrained to slope –1.0 [9]. p*A*_2_ is the positive value of the intercept of the line derived by plotting log (DR – 1) *vs* log [antagonist]. The log (DR – 1) was calculated from three different antagonist concentrations, and each concentration was tested from four to six times. Dose-ratio (DR) values represent the ratio of the potency of the agonist carbachol (EC_50_) in the presence of the antagonist and in its absence. Parallelism of concentration–response curves was checked by linear regression, and slopes were tested for significance (*P* < 0.05).

**Table S7E.Comparison of the significance values of Table S7D**

PREVENTION

| **Gallbladder** | CCh | CD *vs* Ph | Not significant |
| --- | --- | --- | --- |
|  | Atropine | CD *vs* Ph | Not significant |
| **Ileum** | CCh | CD *vs* Ph | ***P* < .05** |
|  | Atropine | CD *vs* Ph | Not significant |
| **Distal colon** | CCh | CD *vs* Ph | **P < .05** |
|  | Atropine | CD *vs* Ph | Not significant |

**INDUCTION**

| **Gallbladder** | CCh | CD *vs* Ph | ***P* < .05** |
| --- | --- | --- | --- |
|  | Atropine | CD *vs* Ph | ***P* < .05** |
| **Ileum** | CCh | CD *vs* Ph | Not significant |
|  | Atropine | CD *vs* Ph | Not significant |
| **Distal colon** | CCh | CD *vs* Ph | Not significant |
|  | Atropine | CD *vs* Ph | Not significant |

**REMISSION**

| **Gallbladder** | CCh | CD *vs* Ph | ***P* < .05** |
| --- | --- | --- | --- |
|  | Atropine | CD *vs* Ph | ***P* < .05** |
| **Ileum** | CCh | CD *vs* Ph | ***P* < .05** |
|  | Atropine | CD *vs* Ph | ***P* < .05** |
| **Distal colon** | CCh | CD *vs* Ph | ***P* < .05** |
|  | Atropine | CD *vs* Ph | ***P* < .05** |
