# Supplementary material for: Antiinflammatory Effect of Phytosterols in Experimental Murine Colitis Model: Prevention, Induction, Remission Study
Source: PLoS One. 2014 Sep 30;9(9):e108112. doi: 10.1371/journal.pone.0108112 (PMC4182327; doi:10.1371/journal.pone.0108112)
Supplement: File S8 — Supporting Additional References. (DOC) [file pone.0108112.s008.doc]

**S8. Supporting Additional References:**

RS1. Moreau RA, Whitaker BD, Hicks KB. (2002) Phytosterols, phytostanols, and their conjugates in foods: structural diversity, quantitative analysis, and health-promoting uses, Prog Lipid Res 41: 457-500.

RS2. Slavin M, Yu LL. (2012) A single extraction and HPLC procedure for simultaneous analysis of phytosterols, tocopherols and lutein in soybeans. Food Chem 135: 2789-2795.

RS3. Hamrouni-Sellami I, Salah HB, Kchouk ME, et al. (2007) Variations in phytosterol composition during the ripening of Tunisian safflower (Carthamus tinctorius L.) seeds. Pak J Biol Sci 10: 3829-3834.

RS4. Reagan-Shaw S, Nihal M, Ahmad N. (2008) Dose translation from animal to human studies revisited. FASEB J 22: 659-661.

RS5. De Smet E, Mensink RP, Plat J. (2012) Effects of plant sterols and stanols on intestinal cholesterol metabolism: suggested mechanisms from past to present. Mol Nutr Food Res 56: 1058-1072.

RS6. Waldner MJ Neurath MF. (2009) Chemically induced mouse models of colitis. Curr Protoc Pharmacol 46:5.55.1-5.55.15.

RS7. Wirtz S, Neufert C, Weigmann B, et al. (2007) Chemically induced mouse models of intestinal inflammation. Nat Protoc 2: 541-546.

RS8. Roda A, Gioacchini AM, Cerrè C, et al. (1995) High-performance liquid chromatographic-electrospray mass spectrometric analysis of bile acids in biological fluids. J Chromatogr B Biomed Appl 665: 281-294.

RS9. Tallarida RJ, Murray RB. (1957) Manual of Pharmacologic Calculations with Computer Programs, 2nd ed.; Springer-Verlag: New York.

RS10. Arunlakshana D, Schild HO. (1959) Some Quantitative Uses of Drug Antagonists. Br J Pharmacol 14: 48-58.
